# Impact of Race and Health Insurance Status on Response to Neoadjuvant Chemotherapy for Breast Cancer Patients

**DOI:** 10.7759/cureus.16127

**Published:** 2021-07-02

**Authors:** Yvonne Ho, Alexander Harris, Michael Wesolowski, Tamer Refaat, William Small, Tarita O Thomas

**Affiliations:** 1 Radiation Oncology, Loyola University Chicago Stritch School of Medicine, Maywood, USA; 2 Biostatistics, Loyola University Chicago Stritch School of Medicine, Maywood, USA; 3 Radiation Oncology, Henry Ford Health System, Detroit, USA

**Keywords:** neoadjuvant chemotherapy, breast cancer, response rate, race, insurance

## Abstract

We evaluated how race, insurance status, and other sociodemographic, tumor, and treatment variables influenced the response to neoadjuvant chemotherapy (NAC) in breast cancer. We performed an IRB-approved retrospective review of 298 breast cancer patients treated with NAC from 2006-2018 at our institution. Univariable and multivariable binary logistic regression analyses were performed to estimate the effects of race, insurance status, and other variables on outcomes. Outcomes of interest included pathologic complete response (pCR), partial response (pPR), and any response (pCR or pPR). Sixty-nine patients (23%) identified as African American. One hundred sixty-eight (57%) patients had private insurance, 71 (24%) had Medicare, 40 (14%) had Medicaid, and 17 (6%) had no insurance. Insurance status was a predictor for any clinical response to NAC in both univariable and multivariable analyses (*p*<0.01), where odds of pCR or pPR were lower for patients with Medicare compared to private insurance (OR 0.32, 95% CI: 0.15-0.70, *p*<0.01). Other variables significant for the response to NAC included body mass index, hormone receptor status, clinical group stage, and Ki-67. Race did not influence the response to NAC. Insurance provider, body mass index, hormone receptor status, clinical group stage, and Ki-67 may be useful predictors of treatment outcomes. Future studies that assess the impacts of insurance status and other identified factors on treatment response may help evaluate outcomes in at-risk populations with factors that preclude full benefit from NAC.

## Introduction

Despite the general improvement in breast cancer (BC) survival, racial inequalities in BC mortality rates and outcomes continue. African American (AA) women still experience an age-adjusted mortality rate that is upwards of 40% higher than white patients [1,2]. Differences in survival rates have also been associated with health insurance status, where Medicaid-insured and uninsured patients present with more unfavorable oncologic characteristics and higher mortality rates compared to privately insured patients [3]. Various patient, tumor, and treatment-specific variables have been studied in an attempt to explain this disparity. However, the majority of these studies were conducted in the setting of adjuvant chemotherapy and not neoadjuvant chemotherapy (NAC).

It is unclear whether these survival disparities continue to exist among patients receiving NAC, with some reports noting lower rates of pathologic complete response (pCR), defined as no residual cancer in the breasts and axillary lymph nodes, in AA patients and others reporting higher rates [4,5]. In this study, we sought to discern the effects of race and health insurance status on BC outcomes among patients who received NAC at a single academic institution. We also aimed to identify other significant predictors of NAC outcomes that may help individualize treatment and optimize patient response.

The abstract of this article was previously presented as a poster presentation at the American Society for Radiation Oncology (ASTRO) Annual Meeting on September 15-18, 2019.

## Materials and methods

Patient population

Information on sociodemographic, tumor, and treatment characteristics was collected by review of electronic medical records from patients with BC treated with NAC and adjuvant radiation therapy at Loyola University Medical Center from 2006 to 2018. The institutional review board of Loyola University Chicago Health Sciences Division issued approval number LU210852. Patient characteristics included age at diagnosis, race, body mass index (BMI), diabetes mellitus (DM), and health insurance status. Tumor characteristics included cancer staging before and after treatment, hormone receptor (HR) status, cancer histology, cancer grade, BRCA gene status, Ki-67, extracapsular extension (ECE), and lymphovascular invasion (LVI). Treatment characteristics included chemotherapy duration and regimen. Outcomes of interest included rates of pCR, pPR (partial response, defined as cancer downstaging without complete response), and any positive response (pCR or pPR).

Statistical analysis

Frequencies and percentages are reported to describe categorical variables. Univariable binary logistic regression analysis was used to estimate the unadjusted effects of predictors on study outcomes. Predictors demonstrating significant unadjusted effects with p<0.05 in univariable analyses were considered for final multivariable models in addition to race. Multivariable binary logistic regression analysis was used to estimate the independent effects of predictors after adjusting for race, BMI, insurance status, HR status, Ki-67, and cancer group stage. Fisher’s exact test was used to evaluate the associations of predictors with study outcomes where precise, reliable odds ratios could not be obtained. Statistical significance was determined at an α<0.05 level. All analysis was conducted using SAS 9.4 (SAS Institute, Cary, NC, USA).

## Results

Two hundred ninety-eight patients were included in the study with 69 (23.3%) patients identifying as AA. One hundred sixty-eight (56.8%) patients had private insurance while 71 (24.0%) had Medicare, 40 (13.5%) had Medicaid, 17 (5.7%) had no insurance, and two were not reported. Baseline sociodemographic, tumor, and treatment characteristics are shown in Table 1. A total of 188 (68.4%) patients had a response to NAC, with 77 (28.0%) attaining pCR and 111 (40.4%) attaining pPR. Forty-four (16.0%) patients had stable disease while 43 (15.6%) patients had disease progression.

**Table 1:** Patient baseline demographic and clinical characteristics BMI, body mass index; HR, hormone receptor; ER, estrogen receptor; PR, progesterone receptor; HER2, human epidermal growth factor receptor 2; BRCA, breast cancer gene; ECE, extracapsular extension; LVI, lymphovascular invasion; AC-T, Doxorubicin + Cyclophosphamide + Paclitaxel; TC, Paclitaxel + Cyclophosphamide; PTCH, Pertuzumab + Paclitaxel + Carboplatin + Trastuzumab; PTH, Paclitaxel + Carboplatin + Trastuzumab.

Variable	Frequency, n (%)
Age (years)	
66+	47 (15.8)
51-65	131 (44.0)
36-50	101 (33.9)
<35	19 (6.4)
Race	
Black	69 (23.3)
Hispanic	27 (9.1)
Other	19 (6.4)
White	181 (61.2)
BMI	
>35	53 (18.5)
30.1-35	76 (26.5)
25.1-30	77 (26.8)
<25	81 (28.2)
Diabetes Mellitus	
Diabetes	47 (15.8)
No Diabetes	251 (84.2)
Insurance Provider	
Uninsured	17 (5.7)
Medicare	71 (24.0)
Medicaid	40 (13.5)
Private	168 (56.8)
cT Stage	
T1(A-C)	39 (13.8)
T2	150 (53.0)
T3	61 (21.6)
T4(A-D)	33 (11.7)
cN Stage	
N0	105 (37.8)
N1	126 (45.3)
N2(A-B)	24 (8.6)
N3(A-C)	23 (8.3)
yp T Stage	
T0/TIS	99 (34.6)
T1(A-C)	109 (38.1)
T2	47 (16.4)
T3	21 (7.3)
T4(A-D)	10 (3.5)
yp N Stage	
pN0	158 (55.6)
pN1(A-C, mic)	72 (25.4)
pN2(A-B)	31 (10.9)
pN3(A-C)	23 (8.1)
Group Stage	
Stage I	11 (4.0)
Stage II	168 (61.5)
Stage III	86 (31.5)
Stage IV	8 (2.9)
HR Status	
ER-/PR-	109 (37.0)
ER-/PR+	7 (2.4)
ER+/PR-	42 (14.2)
ER+/PR+	137 (46.4)
HER2 Status	
HER2+	78 (26.7)
HER2-	214 (73.3)
Histology	
Mixed/Poorly Differentiated/Other	9 (3.1)
Lobular	20 (6.8)
Ductal	266 (90.2)
Grade	
Grade 1	16 (5.5)
Grade 2	89 (30.7)
Grade 3	185 (63.8)
BRCA Status	
BRCA+	11 (9.7)
BRCA-	102 (90.3)
Ki-67	
Low (<10)	29 (11.7)
Intermediate (10-20)	34 (13.7)
High (>20)	186 (74.7)
ECE	
ECE+	64 (45.7)
ECE-	76 (54.3)
LVI	
LVI+	76 (33.2)
LVI-	153 (66.8)
Chemotherapy Duration	
<8 weeks	10 (4.0)
8-12 weeks	30 (11.9)
12-16 weeks	66 (26.1)
>16 weeks	147 (58.1)
Chemotherapy Regimen	
AC-T	150 (52.3)
TC	4 (1.4)
PTCH/PTH	26 (9.1)
Other	107 (37.3)

Results from univariable and multivariable analyses are reported in Table 2 and Table 3, respectively. While DM, insurance provider, HR and HER2 status, grade, ECE, LVI, and chemotherapy duration significantly influenced the rate of pCR in the univariable analysis, only Ki-67, BMI, and HR status remained significant following adjusted analysis where triple-negative breast cancer (TNBC) was associated with improved outcomes.

**Table 2:** Univariable analysis of sociodemographic, tumor, and treatment variables on treatment response * Statistically significant at α<0.05 level †Fisher’s Exact Test p-value BMI, body mass index; BRCA, breast cancer gene; NA, not available; HR, hormone receptor; ER, estrogen receptor; PR, progesterone receptor; HER2, human epidermal growth factor receptor 2; ECE, extracapsular extension; LVI, lymphovascular invasion; AC-T, Doxorubicin + Cyclophosphamide + Paclitaxel; TC, Paclitaxel + Cyclophosphamide; PTCH, Pertuzumab + Paclitaxel + Carboplatin + Trastuzumab; PTH, Paclitaxel + Carboplatin + Trastuzumab.

	pCR			pPR	
Patient Variable	OR (95% CI)		p-value	OR (95% CI)	p-value
Age (years)					
66+	0.67 (0.15-3.05)		0.60	1.22 (0.38-3.93)	0.74
51-65	1.96 (0.53-7.28)		0.31	0.84 (0.29-2.48)	0.76
36-50	2.10 (0.56-7.92)		0.27	1.60 (0.54-4.75)	0.40
<35 (ref)					
Race					
Black	1.41 (0.77-2.59)		0.27	0.89 (0.50-1.58)	0.69
Non-Black (ref)					
BMI					
>35	1.03 (0.46-2.30)		0.95	0.69 (0.34-1.42)	0.32
30.1-35	1.03 (0.50-2.15)		0.93	0.38 (0.19-0.74)	0.01*
25.1-30	1.49 (0.72-3.06)		0.28	0.57 (0.29-1.12)	0.10
<25 (ref)					
Diabetes Mellitus					
Diabetes	0.41 (0.17-0.96)		0.04*	1.44 (0.76-2.73)	0.26
No Diabetes (ref)					
Insurance Provider					
Uninsured	0.51 (0.14-1.89)		0.31	2.13 (0.72-6.29)	0.17
Medicare	0.45 (0.22-0.90)		0.03*	0.79 (0.44-1.44)	0.44
Medicaid	0.86 (0.40-1.88)		0.71	0.87 (0.41-1.81)	0.70
Private (ref)					
BRCA Status					
BRCA+	0.56 (0.11-2.86)		0.49	0.76 (0.18-3.21)	0.70
BRCA- (ref)					
Tumor Variable					
cT Stage					
T1(A-C) (ref)				NA	<0.01*
T2	1.04 (0.48-2.25)	0.92			
T3	0.57 (0.22-1.45)	0.24			
T4(A-D)	0.32 (0.09-1.13)	0.08			
cN Stage					
N0 (ref)					
N1	0.81 (0.46-1.45)	0.48		1.65 (0.95-2.86)	0.08
N2(A-B)	0.61 (0.21-1.78)	0.36		2.98 (1.18-7.52)	0.02*
N3(A-C)	0.88 (0.31-2.46)	0.80		2.52 (0.97-6.54)	0.06
Group Stage					
Stage I (ref)				NA	<0.01*†
Stage II	1.25 (0.32-4.91)	0.75			
Stage III	0.83 (0.20-3.45)	0.80			
Stage IV	0.38 (0.03-4.55)	0.45			
HR Status					
ER-/PR-	3.75 (2.04-6.88)	<0.01*		0.66 (0.39-1.12)	0.12
ER-/PR+	2.50 (0.43-14.54)	0.31		0.61 (0.11-3.43)	0.57
ER+/PR-	1.55 (0.64-3.75)	0.33		0.79 (0.38-1.65)	0.53
ER+/PR+ (ref)					
HER2 Status					
HER2+	2.19 (1.24-3.87)	0.01*		1.20 (0.70-2.06)	0.51
HER2- (ref)					
Histology					
Mixed/Poorly Differentiated/Other	0.14 (0.01-1.10)	NA		2.53 (0.59-10.84)	0.21
Lobular	0.19 (0.02-0.75)			1.11 (0.43-2.85)	0.84
Ductal (ref)					
Grade					
Grade 1 (ref)	NA	<0.01*†			
Grade 2				0.97 (0.30-3.14)	0.96
Grade 3				0.45 (0.14-1.39)	0.16
Ki-67					
Low (<10)	0.04 (0.01-0.27)	NA		2.21 (0.97-5.00)	0.06
Intermediate (10-20)	0.31 (0.10-0.82)			1.37 (0.64-2.95)	0.42
High (>20) (ref)					
ECE					
ECE+	0.10 (0.01-0.80)	0.03*		0.72 (0.36-1.45)	0.36
ECE- (ref)					
LVI					
LVI+	0.28 (0.12-0.66)	<0.01*		0.88 (0.49-1.58)	0.67
LVI- (ref)					
Treatment Variable					
Chemotherapy Duration (Weeks)					
<8	0.33 (0.04-2.82)	0.31		0.36 (0.04-3.12)	0.36
8-12	0.32 (0.10-0.96)	0.04*		1.54 (0.68-3.51)	0.30
12-16	0.90 (0.48-1.70)	0.74		2.19 (1.19-4.02)	0.01*
>16 (ref)					
Chemotherapy Regimen					
AC-T (ref)		NA			
TC	0.38 (0.01-3.67)			1.18 (0.16-0.58)	0.87
PTCH/PTH	2.51 (1.05-5.92)			1.01 (0.43-2.34)	0.99
Other	1.63 (0.91-2.91)			0.51 (0.30-0.88)	0.02*

**Table 3:** Multivariable analysis for adjusted effects of variables on treatment response * Statistically significant at α<0.05 level HR, hormone receptor; BMI, body mass index; ER, estrogen receptor; PR, progesterone receptor; HER2, human epidermal growth factor receptor 2; NA, not available.

	pCR		pPR		Any Positive Response (pCR or pPR)	
Variable	OR (95% CI)	p-value	OR (95% CI)	p-value	OR (95% CI)	p-value
Race						
Black	0.84 (0.38-1.86)	0.67	1.30 (0.60-2.85)	0.51	1.19 (0.52-2.71)	0.68
Non-Black (ref)						
BMI						
>35	1.68 (0.57-4.92)	0.35	0.31 (0.12-0.81)	0.02*	0.45 (0.16-1.25)	0.13
30.1-35	1.73 (0.65-4.59)	0.27	0.13 (0.05-0.33)	<0.01*	0.20 (0.08-0.51)	<0.01*
25.1-30	2.64 (1.00-6.94)	0.05*	0.33 (0.13-0.81)	0.02*	0.70 (0.27-1.78)	0.45
<25 (ref)						
Insurance Provider						
Uninsured	0.41 (0.08-2.18)	0.30	2.40 (0.65-8.78)	0.19	1.25 (0.29-5.31)	0.76
Medicare	0.46 (0.20-1.10)	0.08	0.65 (0.30-1.40)	0.27	0.32 (0.15-0.70)	<0.01*
Medicaid	0.61 (0.21-1.73)	0.35	0.82 (0.30-2.25)	0.70	0.44 (0.16-1.18)	0.10
Private (ref)						
HR Status						
Other	3.29 (1.19-9.09)	0.02*	0.86 (0.37-1.97)	0.72	1.79 (0.79-4.04)	0.16
ER-/PR-/HER2-	4.14 (1.51-11.33)	0.01*	1.25 (0.51-3.02)	0.63	3.63 (1.49-8.85)	<0.01*
ER-/PR-/HER2+	NA	NA	0.37 (0.10-1.32)	0.13	NA	NA
ER+/PR+/HER2- (ref)						
Ki-67						
Low (<10)	0.26 (0.08-0.81)	0.02*	3.23 (1.08-9.60)	0.04*	1.02 (0.35-2.95)	0.98
Intermediate (10-20)	0.26 (0.08-0.81)	0.02*	1.30 (0.49-3.44)	0.59	0.70 (0.26-1.85)	0.47
High (>20) (ref)						
Group Stage						
Stage IV	0.26 (0.02-2.79)	0.26	NA	NA	NA	NA
Stage III	0.65 (0.27-1.57)	0.34	NA	NA	5.75 (2.34-14.16)	<0.01*
Stage IIB	0.78 (0.35-1.72)	0.54	3.73 (1.65-8.42)	<0.01*	2.64 (1.24-5.66)	0.01*
Stage I-IIA (ref)						

BMI, cT and cN staging, clinical group stage, and chemotherapy duration and regimen significantly influenced pPR in the univariable analysis. After adjusted analysis, significance persisted only for Ki-67, BMI, and clinical group stage. BMI, insurance provider, HR status, and clinical group stage were also significant predictors of any positive response to NAC following adjusted analysis.

There were no significant associations between race and pCR (33.3% AA vs. 26.2% non-AA, p=0.27), pPR (38.1% vs. 41.0%, p=0.69), or any clinical response to NAC (71.4% vs 67.1%, p=0.52) (Table 4). Race did not influence any outcome in the univariable analysis and in the multivariable analysis, even after adjusting for insurance provider and other variables.

**Table 4:** Effect of race (AA vs. non-AA) on treatment response

Treatment Response	Unadjusted OR (95% CI)	p-value	Insurance Provider-Adjusted OR (95% CI)	p-value
pCR	1.41 (0.77-2.59)	0.27	1.49 (0.80-2.75)	0.21
pPR	0.89 (0.50-1.58)	0.69	0.91 (0.51-1.62)	0.74
Any Response (pCR or pPR)	1.22 (0.66-2.27)	0.52	1.33 (0.70-2.51)	0.38

However, patients with Medicare had lower odds of pCR (OR 0.45, 95% CI: 0.22-0.90, p=0.03) or any positive response to NAC (OR 0.40, 95% CI: 0.22-0.74, p<0.01) compared to patients with private health insurance. This Medicare effect remained significant for any positive response to NAC after adjusted analysis (53.7% vs 74.2%, p<0.01). There was also no difference in the chemotherapy regimen used as a function of race (p=0.29) or insurance status (p=0.18).

## Discussion

This study aimed to assess the impact of AA race and insurance status on response to NAC. Our study showed that insurance providers, as well as BMI, HR status, clinical group stage, and Ki-67, influenced the response to NAC in women with BC. However, our data did not show that AA race significantly influenced this result, even after controlling for the potential confounding influence of insurance status. We did find a decreased response to NAC in patients with Medicare compared to those with private insurance, which persisted on multivariable analysis.

Response to NAC is impactful to both women with BC and their oncology team as it can dictate subsequent management options and prognosis. A positive response to NAC can downstage a woman’s BC and allow for breast conservation therapy as opposed to mastectomy [6,7]. Response to NAC has also been associated with improvements in recurrence-free survival and overall survival, with many studies demonstrating pCR as a strong predictor of outcomes [8,9].

The influence of insurance status on BC management and survival is well-documented. Multiple publications have found reduced cancer-specific and overall survival in women with no insurance or Medicaid. Other studies have found differences in rates and types of surgery, radiation, and systemic therapy utilization as a function of insurance status [10-12]. In reviewing the care of over 4,500 women with BC, Ayanian et al. found that women with Medicaid or no insurance had a worse overall survival with a risk of death 40% and 49% higher, respectively. This was thought to be due to these women presenting with more advanced disease than their privately insured counterparts [10]. In a review of the National Cancer Database (NCDB), Riba et al. found that uninsured women were less likely to have breast conservation therapy or to undergo breast reconstruction after surgery [11]. For these reasons, patients with no or insufficient insurance could benefit from NAC.

However, our results showed that women without private health insurance had a lower likelihood of achieving either a pCR or pPR after NAC. The etiology of this difference is likely multifactorial. For one, women without sufficient medical insurance may not utilize ancillary medical services such as psychological therapy, nutrition courses, or exercise programs that have been shown to improve BC outcomes [13-15]. Additionally, health insurance may be a proxy for other socioeconomic factors such as income, medical insight, access to care, and nutritional status that can influence tolerance of chemotherapy and overall BC outcomes [16-18].

Race also has a well-documented influence on BC management and outcomes. In a review of the NCDB, Killelea et al. showed that NAC was given to AA patients at a higher rate than white patients. They attributed this to AA patients presenting with more advanced tumors and a greater proportion of AA patients presenting with TNBC and HER2+ tumors. While they found no difference between pCR rates in white and non-white women with ER+ or PR+ tumors, they noted a lower pCR rate in AA women with ER-/PR-/HER2+ cancers (43% v 54%, p=0.001) and TNBC (37% v 43%, p<0.001) compared to white women. This difference persisted when accounting for insurance status [19]. In contrast, through a retrospective review at the University of Virginia, Knisely et al. found that race did not influence the pCR rate between white and AA women (37% vs 21%, p=0.08). They did, however, find that race influenced the rate of NAC completion, with white women more often completing the recommended course of NAC than AA women (76% vs 50%, p=0.006) [20]. Similarly, in a review of MD Anderson patients, Chavez-MacGregor et al. found no difference in pCR rates between AA and white women (12.3% in both, p=0.788) [21]. Our study mirrors the results of these two single-institution studies in finding no difference in pCR rates between AA and white women. While our study did not look at delays in NAC treatment or completion rates, chemotherapy duration and regimen did not significantly influence outcomes.

The single-institution nature of our study led to a relatively small sample size, which may limit the generalizability of results. With a larger multi-institutional study, the pooling of data in a meta-analysis, or a prospective study design, a more complete amount of data would be available to help identify additional factors that influence clinical outcomes after NAC in BC. Our study also did not evaluate treatment-related toxicities or chemotherapy completion rates as a function of race or insurance status. We plan to explore these topics in future research for a more complete evaluation of therapy efficacy.

## Conclusions

Racial and socioeconomic disparities in BC survival rates continue to be investigated among patients who received NAC. This study assessed the effects of race, health insurance status, and other clinically relevant variables on outcomes such as pCR at a single academic institution. While race did not significantly affect outcomes in this study, patients with Medicare had a decreased response to NAC compared to those with private health insurance. Future studies should assess the impact of race, insurance status, and other identified factors on clinical response to NAC for BC in a multi-institutional, prospectively collected manner.
